# Early‐onset vitamin B_6_‐dependent epilepsy due to pathogenic *PLPBP* variants in a premature infant: A case report and review of the literature

**DOI:** 10.1002/jmd2.12183

**Published:** 2020-11-15

**Authors:** Oliver Heath, James Pitt, Simone Mandelstam, Carl Kuschel, Anand Vasudevan, Sarah Donoghue

**Affiliations:** ^1^ Department of Metabolic Medicine The Royal Children's Hospital Melbourne Australia; ^2^ Department of Biochemical Genetics, Victorian Clinical Genetics Service Murdoch Children's Research Institute Melbourne Australia; ^3^ Department of Medical Imaging The Royal Children's Hospital and Murdoch Children's Research Institute Melbourne Australia; ^4^ Department of Obstetrics and Gynecology The Royal Women's Hospital Melbourne Australia; ^5^ Department of Genetics The Royal Women's Hospital Melbourne Australia; ^6^ Department of Paediatrics University of Melbourne Melbourne Australia; ^7^ Department of Radiology University of Melbourne Melbourne Australia

**Keywords:** *PLPBP*, PLPHP, prematurity, *PROSC*, pyridoxal‐5′‐phosphate, pyridoxine, vitamin B_6_‐dependent epilepsy

## Abstract

Vitamin B_6_‐dependent epilepsies are a heterogeneous group of disorders characterized by decreased availability of the active cofactor pyridoxal‐5′‐phosphate (PLP). While pathogenic variants in *ALDH7A1* or *PNPO* genes account for most cases of these disorders, biallelic pathogenic variants in *PLPBP* have been shown to cause a form of early onset vitamin B_6_‐dependent epilepsy (EPVB6D). PLPBP is thought to play a role in the homeostatic regulation of vitamin B_6_, by supplying PLP to apoenzymes while limiting side‐reaction toxicity related to excess unbound PLP. Neonatal‐onset intractable seizures that respond to pyridoxine and/or PLP are a predominant feature of EPVB6D in humans. Unlike other causes of vitamin B_6_‐dependent epilepsies; however, a specific biomarker for this disorder has yet to be identified. Here we present data from a premature infant found to have pathogenic variants in *PLPBP* and propose that prematurity may provide an additional clue for early consideration of this diagnosis. We discuss these findings in context of previously published genotypic, phenotypic, and metabolic data from similarly affected patients.

**Synopsis**

Biallelic *PLPBP* variants are an important cause of vitamin B_6_‐dependent epilepsy, and in the absence of a specific diagnostic biochemical profile, prematurity may provide an additional clue for early diagnosis of EPVB6D in infants presenting with seizures within the first 24 hours of life.

**Abbreviations**
AADC: aromatic l‐amino decarboxylase
ATQ: antiquitin
CSF: cerebrospinal fluid
EPVB6D: early‐onset vitamin B_6_‐dependent epilepsy
HVA: homovanillic acid
IVH: intraventricular hemorrhage
P6C: Δ^1^‐piperideine‐6‐caboxylate
PA: pyridoxic acid
PLP: pyridoxal‐5′‐phosphate
PLPBP: PLP‐binding protein
PM: pyridoxamine
PN: pyridoxine
PNPO: pyridox(am) ine 5′‐phosphate oxidase
WES: whole‐exome sequencing
α‐AASA: α‐aminoadipic semialdehyde

## 1 INTRODUCTION

Pyridoxal‐5′‐phosphate (PLP) is the bioactive form of vitamin B_6_ and functions as a prosthetic group in more than 140 catalytic reactions, representing >4% of all classified enzymatic activities.^1^ Vitamin B_6_ exerts critical coenzymatic activity in several pathways including amino acid and neurotransmitter metabolism, glycogenolysis and hemoglobin synthesis and function.^2^

Many PLP‐dependent enzymes have important roles within the central nervous system, so it is unsurprising that disorders in B_6_ metabolism often result in seizure disorders. Antiquitin (ATQ) deficiency (OMIM #266100) and pyridox(am)ine 5′‐phosphate oxidase (PNPO) deficiency (OMIM #610090) are the most prevalent B_6_‐dependent epilepsies, resulting in inactivation and reduced formation of PLP, respectively.^3^ Both ATQ deficiency and PNPO deficiency are recessively inherited disorders, caused by pathogenic variants in *ALDH7A1* and in *PNPO*, respectively. Other vitamin B_6_‐responsive seizure disorders include congenital hypophosphatasia (OMIM #241500) due to pathogenic *ALPL* variants, hyperprolinaemia type II (OMIM #239510) caused by pathogenic variants in *ALDH4A1*, glycosylphosphatidylinositol anchor defects and molybdenum cofactor deficiencies (OMIM #252150 and #252160).^2^

Early‐onset vitamin B_6_‐dependent epilepsy (EPVB6D; OMIM #617290) due to autosomal recessive pathogenic variants in *PLPBP* was recently discovered.^4^, ^5^ *PLPBP* encodes an evolutionarily conserved protein involved in PLP‐binding. Studies on its bacterial orthologues suggested a role of this protein family in vitamin B_6_ homeostasis, supplying PLP to apoenzymes while minimizing the toxic effects of the PLP aldehyde.^4^, ^6^, ^7^ Involvement of PLPBP in mitochondrial metabolism has also been proposed in yeast.^8^ Finally, *Plpbp*‐null zebrafish provided the first animal model organism for EPVB6D, with larvae developing spontaneous seizure‐like behavior responsive to PLP and pyridoxine.^8^

Forty‐four cases of EPVB6D have been reported in humans,^4^, ^5^, ^8^, ^9^, ^10^, ^11^, ^12^, ^13^ with a clinical picture dominated by neonatal‐onset intractable seizures responsive to pyridoxine and/or PLP, microcephaly, structural brain abnormalities and lactic acidosis. Unlike other causes of B_6_‐dependent epilepsies, there is no specific biomarker for this disorder.^5^

We report a premature infant with vitamin B_6_‐dependent epilepsy caused by pathogenic biallelic variants in *PLPBP* and consider prematurity as another possible clue to the early diagnosis of EPVB6D in neonates seizing within the first day of life. Findings from previously described patients with EPVB6D are also summarized according to available genotypic, phenotypic and biochemical information.

## 2 CASE REPORT

We report a 6‐month‐old (4 months corrected) female infant who was born at 34 + 4 weeks gestation following premature rupture of membranes and fetal bradycardia. She is the second of two children born to nonconsanguineous Australian parents with no family history of epilepsy or developmental delay. Her APGAR scores at birth were normal, birthweight was 2340 g (Fenton chart: 50th‐90th centile), length was 44.5 cm (50th centile) and head circumference was 30 cm (10th‐25th centile). She developed tonic and generalized tonic‐clonic seizures and hypoglycaemia within 4 hours of life requiring transfer to a tertiary care NICU. Her seizures were refractory to treatment with phenobarbitone (day 1‐2), midazolam (day 3) and levetiracetam (day 3). She developed apnea and required intubation on day 2 and EEG demonstrated a burst‐suppression pattern.

**FIGURE 1:**
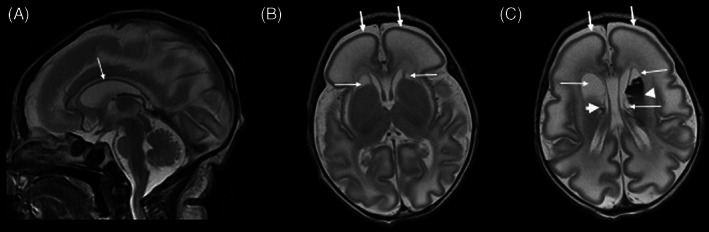
Brain magnetic resonance imaging in our patient day 6 day of life (corrected gestational age 35^+3^weeks). A, Sagittal T2 weighted image demonstrates small cerebrum and cerebellum with very thin corpus callosum. B and C, Axial T2 weighted images demonstrate simplified gyral pattern with thin lissencephalic cortex more severe in the frontal lobes (thick arrows), decreased white matter volume with markedly abnormal T2 hyperintensity, bilateral periventricular cysts (thin arrows) with blood products in the large left sided cyst and in a tiny right sided cyst (arrowheads). Prominent ventricles and large extra‐axial CSF spaces in keeping with small brain

Day 1 cranial ultrasound showed bilateral intraventricular hemorrhages (IVH), with presence of fibrin stranding suggestive of an antenatal bleed. MRI brain at corrected age of 35 + 3 weeks demonstrated a small brain with abnormally T2 hyperintense white matter, decreased white matter volume and thin corpus callosum. Cortex was thin with frontal lissencephaly and simplified gyral pattern posteriorly. There were large bilateral thin walled subependymal/periventricular cysts, some of which contained blood products (Figure 1). Her septic screen was normal. Molecular karyotype showed a maternally‐inherited heterozygous 140 kb deletion in chromosome region 19p13.3 involving the *APC2* gene.

Empiric therapy with pyridoxine (30 mg/kg/day), folinic acid (7.5 mg/day) and biotin (10 mg/day) was commenced on day 5. Her clinical seizures improved after the first dose of pyridoxine, enabling her to be successfully extubated on day 7 of life. Treatment was later rationalized based on findings of normal urine Δ^1^‐piperideine‐6‐caboxylate (P6C), and PLP (30 mg/kg/day) was commenced on day 9 in addition to levetiracetam (20 mg/kg/day). Genetic testing subsequently confirmed a diagnosis of EPVB6D.

At 6 months of age, she remains seizure‐free on PLP and a weaning dose of levetiracetam. She has acquired microcephaly—head circumference 39.5 cm (<3rd centile). Her length and weight continue to track normally. She has no dysmorphic features. There are no concerns with her hearing or vision, and her motor and speech developmental milestones are age‐appropriate.

## 3 METHODS

### 3.1 Biochemical analyses

Pretreatment blood, urine and CSF samples were collected and processed for routine biochemistry, amino acids, neurotransmitters, organic acids, P6C and pipecolic acid according to standard protocols. Other urine metabolites were measured by flow‐injection tandem mass spectrometry using a panel of targeted MRMs as previously described.^14^

### 3.2 Next‐generation sequencing analysis

Whole exome sequencing (WES) was performed on DNA isolated from blood using massively parallel sequencing (Agilent SureSelectXT Low Input enzymatic fragmentation kit, CREv2, Illumina Sequencers) with a targeted mean coverage of 100×. Data processing, including read alignment to the reference genome (GRCh38) and variant calling, was carried out using Cpipe.^15^ Variant analysis and interpretation within the target region (coding exons ±8 bp) was performed using Agilent Alissa Interpret. Curation of variants was phenotype‐driven with custom gene (*APC2*) and precurated gene lists (https://panelapp.agha.umccr.org/) used for variant prioritization: Intellectual disability syndromic and nonsyndromic v0.2038, Lissencephaly and Band Heterotopia v0.21, Neurotransmitter Defects v0.1, Mitochondrial disease v0.79.

## 4 RESULTS

Routine lab studies were notable for metabolic acidosis (pH 7.25) and hyperlactatemia (peak of 11.8 mmol/L at 12 hours; reference range [RR]: 1.0‐1.8 mmoL/L) which slowly normalized by day 6. Pyruvate was also high (0.27 mmol/L; RR: 0‐0.1 mmol/L). Her pretreatment amino acid profile demonstrated elevated glycine 1024 μmol/L (RR: 106‐254 μmol/L) and alanine 585 μmol/L (RR: 132‐455 μmol/L). A microcytic anemia was present (Hb 104 g/L; RR: 145‐225 g/L).

Pretreatment CSF screening on day 5 revealed high lactate 4.3 mmol/L (RR: 1.2‐2.3 mmol/L); high pyruvate 0.16 mmol/L (RR: 0.06‐0.13 mmol/L), and low neurotransmitter levels: HVA 0.47 μmol/L (RR: 0.54‐1.14 μmol/L) and 5‐HIAA 0.32 μmol/L (RR: 0.38‐1.03 μmol/L). IVH‐related protein contamination made the CSF amino acid profile uninterpretable.

Metabolic screening of urine on day 6, 24 hours after commencement of pyridoxine therapy showed normal levels of P6C and pipecolic acid; elevated vanillactic acid 13 μmol/mmol creatinine (RR: <2 μmol/mmol creatinine); elevated pyridoxic acid, consistent with treatment. Levels of vanillactic acid remained mildly elevated (3 μmol/mmol creatinine μmol/mmol creatinine) after 4 days of vitamin B_6_ treatment. The rest of her metabolic investigations were normal (Supporting Information Table S2).

Trio WES analysis revealed two pathogenic compound heterozygous variants in *PLPBP* on chromosome 8. The paternally inherited variant NM_007198.3(PLPBP):c.207+1G>T; p.(?) is a splicing variant in intron 2. Abnormal splicing was predicted by in silico tools, and the affected nucleotide is highly conserved. The maternally‐inherited missense variant NM_007198.3(PLPBP):c.722G>; p.(Arg241Gln) is located in a conserved motif involved in PLP binding. This variant exists at a frequency of <0.01 in the gnomAD database and was consistently predicted to be damaging by multiple in silico tools. No additional pathogenic variants of interest were detected within the second allele of *APC2*.

## 5 DISCUSSION

This case describes the follow up of a premature infant with early onset vitamin B_6_‐dependent epilepsy caused by pathogenic biallelic variants in *PLPBP*. Forty‐four other patients with EPVB6D, from 17 different ethnic backgrounds around the world, have previously been reported,^4^, ^5^, ^8^, ^9^, ^10^, ^11^, ^12^, ^13^ (see Supporting Information Tables S1 and S2).

### 5.1 Genotype

Our patient is compound heterozygous for two pathogenic variants in *PLPBP*: c.207+1G>T; p(?) and c.722G>A; p.(Arg241Gln). As summarized in Table 1, 14 missense, 3 nonsense, 2 frameshift, and 3 splice site pathogenic *PLPBP* variants have been reported to date.^4^, ^5^, ^8^, ^9^, ^10^, ^11^, ^12^ Loss‐of‐function is the likely mechanism of disease for this gene, with splice site, nonsense and frameshift mutations often resulting in more severe phenotypes.^8^, ^16^ The splice‐variant c.207+1G>T was described in a Chinese patient with pyridoxine‐dependent epilepsy.^12^ An alternative nucleotide change at the same position, c.207+1G>A, has strong evidence for pathogenicity and has been reported in two other individuals.^4^, ^11^ The second variant, c.722G>A; p.(Arg241Gln), is a missense variant previously reported in two unrelated individuals with EPVB6D and milder phenotypes.^4^, ^5^, ^8^ Functional studies suggest the resulting protein to be less stable and lack PLP binding capacity.^16^

**TABLE 1:** Summary of pathogenic *PLPBP* variants described to date in 33 patients/30 families

Reference	Variant type	Position	cDNA level^a^	Amino acid change	Number of patients		Number of families	
					Homo	Comp Het	Homo	Comp Het
Jiao et al^12^	Nonsense	Exon1	c.45C>A	p.Cys15Ter		1		1
Plecko et al,^5^ Jiao et al^12^	Missense	Exon 1	c.119C>T	p.(Pro40Leu)		2		2
Jensen et al ^11^	Missense	Exon1	c.121C>T	p.(Arg41Trp)	1		1	
Shiraku et al,^9^ Johnstone et al^8^	Missense	Exon 2	c.122G>A	p.(Arg41Gln)	3	1	3	1
Shiraku et al,^9^ Johnstone et al^8^	Missense	Exon 2	c.199G>A	p.(Glu67Lys)	4		4	
Plecko et al^5^	Missense	Exon 2	c.206A>G	p.(Tyr69Cys)	1		1	
Darin et al,^4^ Jensen et al^11^	Splice site	Intron 2	c.207+1G>A	Splicing effect	1	1	1	1
Jiao et al,^12^ this study	Splice site	Intron 2	c.207+1G>T	Splicing effect		2		2
Darin et al^4^	Nonsense	Exon 3	c.211C>T	p.(Gln71Ter)	1		1	
Darin et al^4^	Nonsense	Exon 3	c.233C>G	p.(Ser78Ter)	3		1	
Plecko et al^5^	Frameshift	Exon 4	c.249‐252del	p.(Ser84Cysfs*21)		1		1
Darin et al,^4^ Plecko et al^5^	Missense	Exon 4	c.260C>T	p.(Pro87Leu)	1	1	1	1
Johnstone et al^8^	Missense	Exon4	c.280A>T	p.(Ile94Phe)	1		1	
Jiao et al^12^	Missense	Exon4	c.338T>C	p.(Met113Thr)		1		1
Johnstone et al^8^	Missense	Exon4	c.347C>T	p.(Thr116Ile)	2		2	
Johnstone et al,^8^ Kernohan et al^10^	Frameshift	Exon4	c.370‐373del	p.(Asp124Lysfs*2)	3		3	
Darin et al,^4^ Johnstone et al^8^	Splice site	Intron 4	c.320‐2A>G	Splicing effect		2		2
Darin et al^4^	Missense	Exon 6	c.524T>C	p.(Leu175Pro)	1		1	
Plecko et al,^5^ Shiraku et al^9^	Missense	Exon8	c.614G>A	p.(Arg205Gln)	1	1	1	1
Johnstone et al^8^	Missense	Exon 8	c.671G>C	p.(Gly224Ala)		1		1
Darin et al,^4^ Plecko et al,^5^ this study	Missense	Exon 8	c.722G>A	p.(Arg241Gln)		3		3
Johnstone et al^8^	Missense	Exon 8	c.823C>G	p.(His275Asp)	1		1	

Abbreviations: Comp Het, compound heterozygous; Homo, homozygous; PLPBP, PLP‐binding protein.

^a^ According to RefSeq accession number NM_007198.3. Nucleotide numbering uses +1 as the A of the ATG translation initiation codon in the mRNA reference sequence, with the initiation codon as codon 1.

Our patient was also incidentally found to have a heterozygous deletion at 19p13.3 involving the *APC2* gene. *APC2* is associated with a recessive brain malformation syndrome causing lissencephaly.^17^ The neuroimaging in our patient was not typical for *APC2* pathogenic variants, and WES analysis did not find a second variant.

### 5.2 Phenotype

The frequencies of common clinical features observed in EPVB6 are summarized in Table 2. The phenotype is dominated by neonatal‐onset refractory seizures. Seventy‐eight percent of affected infants had seizures in their first week of life, and in all of those born prematurely, seizures developed within 24 hours of life.^4^, ^8^, ^9^, ^11^ Prematurity has been reported in the PLP and PN responsive epilepsies, presenting in 18% of ATQ deficiency patients and 46% to 50% of patients with PNPO deficiency.^18^, ^19^, ^20^ This also seems to be a feature of EPVB6D with prematurity reported in eight patients (27%), including our patient.^4^, ^8^, ^9^, ^11^ Other pregnancy complications (IUGR,^4^ abnormal movements^4^, ^8^, ^13^) and fetal distress (nonreassuring CTG,^4^, ^8^ meconium stained liquor^4^, ^8^, ^11^) occurred in up to half of all cases. Microcephaly and structural brain abnormalities, including white matter changes, simplified sulcation and periventricular cysts feature prominently and were all present in our case.

**TABLE 2:** Summary of clinical, radiological and metabolic findings in patients with early‐onset vitamin B_6_‐dependent epilepsy caused by pathogenic *PLPBP* variants^a^

	Incidence^b^	Proportion
Clinical features		
Gender		
Male	18/32	56%
Female	14/32	44%
Current age reported	3 m‐30 y	
Consanguinity	20/32	63%
	17 families	
Pregnancy complications	11/32	34%
Fetal distress	10/19	53%
Gestational age^c^		
Preterm	2/30	7%
Late preterm	6/30	20%
Term	22/30	69%
Birth HC centile		
<10%	9/27	33%
10%‐25%	5/27	19%
25%‐50%	8/27	30%
59%‐90%	5/27	19%
Acquired microcephaly	9/16	56%
Seizure onset		
<24 h	19/36	53%
2‐7 d	9/36	25%
1‐4 wk	4/36	11%
>1 m	4/36	11%
Seizure type		
GTC	18/36	50%
Tonic	13/36	36%
Clonic	9/36	25%
Myoclonic	12/36	33%
SIA	9/36	25%
Spasms	5/36	14%
EEG findings		
Burst suppression	16/36	44%
Multifocal spikes	8/36	22%
Focal discharges	5/36	14%
Reduced background activity	5/36	14%
Response to AEDs before B_6_ treatment		
Refractory	25/39	64%
Partial control	13/39	33%
Good control	1/39	3%
Initial B_6_ treatment		
PN	38/41	93%
PLP	2/41	5%
PN + PLP	1/41	2%
Deaths before treatment	4/45	11%
Response to initial B_6_ treatment		
Seizure‐free	31/39	79%
Good control	4/39	10%
Partial control	1/39	3%
No improvement	3/39	8%
B_6_ vitamer switch/response		
PN ➔ PLP	8/32	25%
Seizure‐free	6	
Good control	1	
No improvement	1	
PLP ➔ PN	1/32	3%
No improvement	1	
B_6_ withdrawal	23/41	56%
Seizure relapse/poor response	23/23	100%
Current B_6_ treatment		
PN	12/37	70%
PLP	8/37	22%
Current additional AEDs	16/28	57%
Epilepsy course		
Seizure‐free	17/37	46%
Breakthrough febrile seizures	16/37	43%
Sporadic afebrile seizures	3/37	5%
Photosensitive seizures	1/37	3%
Deaths	5/45	11%
Developmental delay		
Yes/slight	19/38	50%
No	19/38	50%
Intellectual disability	13/20	65%
Brain MRI		
Normal	12/29	41%
Abnormal	17/29	59%
White matter changes	11/17	65%
Simplified sulcation	10/17	59%
Cysts	7/17	41%
Poor PLIC myelination	3/17	18%
Available metabolic profiles^d^		
Urine		
High vanillactic acid	5/20	
High vanilpyruvic acid	1/20	
High *N*‐acetylvanilalanine	1/20	
High lactic acid	2/20	
High pipecolic acid	0/20	
High P6C	0/20	
Normal profile	13/20	
Blood/plasma		
Acidosis	11/26	
High lactate	16/28	
Anemia at birth	3/12	
Amino acids		
– High glycine	10/22	
– High alanine	4/22	
– High threonine	1/22	
– Vitamer profiles (on treatment)	7/7	
– High PLP	4/7	
– High PL	4/7	
– High PA		
CSF		
High lactate	2/6	
Neurotransmitters		
– Low HVA	2/10	
– High 3‐*O*‐methyldopa	3/9	
– High l‐dopa	1/9	
– High 5‐hydroxytryptophan	2/9	
– Normal	5/10	
Amino acids		
– High glycine	7/11	
– High alanine	2/11	
– High threonine	2/11	
– High tryptophan	1/11	
– High tyrosine	1/11	
Homocarnosine		
Undetectable	1/2	
Normal	1/2	
Vitamer profiles		
– Low PLP	2/3	
– Low PL	1/1	

Abbreviations: AED, antiepileptic drug; CSF, cerebrospinal fluid; GTC, generalized tonic‐clonic; HC, head circumference; HVA, homovanillic acid; P6C, Δ^1^‐piperideine‐6‐caboxylate; PA, pyridoxic acid; PL, pyridoxal; PLP, pyridoxal‐5′‐phosphate; PN, pyridoxine; PLIC, posterior limb or internal capsule; SIA, seizures with impaired awareness (lip‐smacking, eye deviation).

^a^ Data compiled from cases described in Darin et al,^4^ Plecko et al,^5^ Johnstone et al,^8^ Shiraku et al,^9^ Jensen et al,^11^ Jiao et al,^12^ Koul et al,^13^ and current study. See Supporting Information Tables S1 and S2 for data extracted from individual studies.

^b^ Number of patients with specific finding/number of patients in whom variable was assessed.

^c^ Gestational age: Preterm <34 weeks; Late preterm 34 + 0 to 36 + 6 weeks; Term 37 + 0 to 40 weeks.

^d^ Unless otherwise specified, results relate to specimens collected before commencement of vitamin B_6_ treatment.

Early treatment with PN or PLP is important as some patients with this disorder have previously died. Four infants died between 2 and 8 weeks of age without receiving B_6_ treatment, while treatment with pyridoxine was introduced late in a patient born at 32 weeks who died at 4 months.^4^, ^8^, ^10^, ^11^ Some of the children who died had been misdiagnosed with a mitochondrial disorder or glycine encephalopathy, before the diagnosis of EPVB6D was made postmortem.^8^, ^11^ Of the five deceased patients with pathogenic *PLPBP* variants, two were born prematurely.^4^, ^8^ Genotype does not seem to be a predisposing factor for lethality, as death occurred in patients with nonsense,^8^ frameshift,^10^ and missense^4^, ^8^, ^11^ variants.

Patients with EPVB6D will respond to PN, PLP, or a combination of both treatments. The majority of patients remained seizure‐free whether on PN or upon switching to PLP, as was observed in our case. An observation that we made from the literature is that 75% of premature infants had their treatment switched from PN to PLP.^4^, ^8^, ^11^ This treatment change was made to treat suspected PNPO deficiency or because of seizure recurrence within the first 2.5 years of life. ^4^, ^8^, ^11^ While additional anticonvulsants were continued in most patients, vitamin B_6_ withdrawal always resulted in seizure recurrence. Half of the patients may experience sporadic or breakthrough seizures.

Neurodevelopmental problems were reported in over half of the patients diagnosed with EPVB6D, with intellectual disability diagnosed in 65%. Some genetic variants such as c.722G>A; p.(Arg241Gln) detected in our case have previously been described in individuals with a milder developmental phenotype including normal intellectual outcome and independence with daily activities.^4^, ^5^

### 5.3 Biochemical profiles

Raised plasma and urinary α‐aminoadipic semialdehyde (α‐AASA) and its equilibrium partner, Δ^1^‐piperideine‐6‐caboxylate (P6C) are characteristic of antiquitin deficiency,^21^ while high plasma pyridoxamine (PM) concentration and high pyridoxamine/pyridoxic acid (PM/PA) ratio show promise as biomarkers for PNPO deficiency,^22^ irrespective of vitamin B_6_ treatment.^20^, ^21^ More specific measurement of PNPO enzyme activity is also available.^23^ Specific biomarkers in EPVB6D, however, have remained elusive to date. Although 44 patients with pathogenic *PLPBP* variants have previously been reported, biochemical data is not consistently available; the available biochemical results are summarized in Table 2.

Findings in our patient were consistent with those previously described and were suggestive of disturbed vitamin B_6_ metabolism. The most common abnormalities observed in blood include elevated lactate (57%) and associated metabolic acidosis (42%). Glycine, the substrate for B_6_‐dependent glycine cleavage system was also elevated (45%). Glycine was also commonly elevated in CSF. Some patients, including our own, display CSF neurotransmitter profiles suggestive of aromatic l‐amino decarboxylase (AADC) deficiency: low homovanillic acid and elevated 3‐*O*‐methyldopa, l‐dopa, and 5‐hydroxytryptophan. These findings are not consistently present, possibly suggestive of the enzyme's sufficient residual activity. Increased lactate and alanine are also not specific for B_6_ deficiency and are frequently increased in other seizure disorders, such as mitochondrial disorders and pyruvate dehydrogenase deficiency. In urine, the most common abnormal finding is raised vanillactic acid (25%), which is another consequence of secondary AADC deficiency. In our patient, urine vanillactic acid was significantly increased on day 6. However, it is notable that most of the biochemical abnormalities secondary to B_6_ deficiency normalize when patients are B_6_ replete. This is illustrated by the near normalization of urine vanillactic acid in our patient after 4 days of treatment, highlighting the importance of obtaining early samples for metabolite testing, preferably before pyridoxine or PLP treatment commences.

Interpretation of B_6_ vitamer profiles is also difficult because of a lack of clearly defined reference ranges. Increased plasma levels of PLP, PL, and PA were reported in a small number of subjects already on B_6_ supplementation.^4^, ^5^ There was no associated accumulation of PN, PM, PNP and PMP, unlike the profiles typically seen in PNPO deficiency.^24^ In CSF, low PLP levels were reported in two patients prior to treatment.^4^ Measurement of intracellular PLP in lysates from patient fibroblasts has yielded incongruent results.^4^, ^8^

In summary, this case highlights the importance of early sample collection and treatment of patients suspected to have with PN or PLP responsive epilepsies. The biochemical profiles in patients with EPVB6D described to date remain nonspecific and may overlap with a wide variety of neonatal presentations, including mitochondrial disease and hypoxic ischemic encephalopathy. Prematurity may provide an additional clue for early diagnosis of this disorder in infants developing seizures in the first 24 hours of life. Additional early clues may include a history fetal distress and microcephaly, although these features are not universally present. Early genetic testing is useful for diagnostic confirmation.

## CONFLICT OF INTEREST

The authors declare that they have no conflict of interest.

## AUTHOR CONTRIBUTIONS

Clinical patient care and diagnosis: Oliver Heath, Carl Kuschel, Anand Vasudevan, Sarah Donoghue. Evaluation/interpretation of metabolic, genetic and radiologic results: Oliver Heath, James Pitt, Simone Mandelstam, Anand Vasudevan, Sarah Donoghue. Drafting and revision of the manuscript: Oliver Heath, Sarah Donoghue, James Pitt, Carl Kuschel, Anand Vasudevan, Simone Mandelstam.

## INFORMED CONSENT

Informed consent was obtained from patient's family for publication of case reports including publication of pictures of cerebral imaging. All procedures followed were in accordance with the Helsinki Declaration of 1975, as revised in 2000.

## Supporting information


**Table S1**. Clinical features of patients with early‐onset vitamin B_6_‐dependent epilepsy caused by variants in *PLPBP*
**Table S2**. Metabolic profiles of patients with *PLPBP* variants described to date

## References

[1] Percudani R , Peracchi A . The B6 database: a tool for the description and classification of vitamin B6‐dependent enzymatic activities and of the corresponding protein families. BMC Bioinform. 2009;10:273. 10.1186/1471-2105-10-273. PMC2748086 19723314

[2] Wilson MP , Plecko B , Mills PB , Clayton PT . Disorders affecting vitamin B6 metabolism. J Inherit Metab Dis. 2019;42:629‐646. 10.1002/jimd.12060. 30671974

[3] Stockler S , Plecko B , Gospe SM Jr , et al. Pyridoxine dependent epilepsy and antiquitin deficiency: clinical and molecular characteristics and recommendations for diagnosis, treatment and follow‐up. Mol Genet Metab. 2011;104:48‐60. 10.1016/j.ymgme.2011.05.014. 21704546

[4] Darin N , Reid E , Prunetti L , et al. Mutations in PROSC disrupt cellular pyridoxal phosphate homeostasis and cause vitamin‐B6‐dependent epilepsy. Am J Hum Genet. 2016;99:1325‐1337. 10.1016/j.ajhg.2016.10.011. 27912044 PMC5142116

[5] Plecko B , Zweier M , Begemann A , et al. Confirmation of mutations in PROSC as a novel cause of vitamin B6‐dependent epilepsy. J Med Genet. 2017;54:809‐814. 10.1136/jmedgenet-2017-104521. 28391250

[6] Prunetti L , El Yacoubi B , Schiavon CR , et al. Evidence that COG0325 proteins are involved in PLP homeostasis. Microbiology. 2016;162:694‐706. 10.1099/mic.0.000255. 26872910

[7] Tremino L , Forcada‐Nadal A , Contreras A , Rubio V . Studies on cyanobacterial protein PipY shed light on structure, potential functions, and vitamin B6‐dependent epilepsy. FEBS Lett. 2017;591:3431‐3442. 10.1002/1873-3468.12841. 28914444

[8] Johnstone DL , Al‐Shekaili HH , Tarailo‐Graovac M , et al. PLPHP deficiency: clinical, genetic, biochemical, and mechanistic insights. Brain. 2019;142:542‐559. 10.1093/brain/awy346. 30668673 PMC6391652

[9] Shiraku H , Nakashima M , Takeshita S , et al. PLPBP mutations cause variable phenotypes of developmental and epileptic encephalopathy. Epilepsia Open. 2018;3:495‐502. 10.1002/epi4.12272. 30525118 PMC6276781

[10] Kernohan KD , Hartley T , Naumenko S , et al. Diagnostic clarity of exome sequencing following negative comprehensive panel testing in the neonatal intensive care unit. Am J Med Genet A. 2018;176:1688‐1691. 10.1002/ajmg.a.38838. 30160830

[11] Jensen KV , Frid M , Stödberg T , et al. Diagnostic pitfalls in vitamin B6‐dependent epilepsy caused by mutations in the PLPBP gene. JIMD Rep. 2019;50:1‐8. 10.1002/jmd2.12063. 31741821 PMC6850975

[12] Jiao X , Xue J , Gong P , et al. Clinical and genetic features in pyridoxine‐dependent epilepsy: a Chinese cohort study. Dev Med Child Neurol. 2020;62:315‐321. 10.1111/dmcn.14385. 31737911

[13] Koul R , Alfutaisi A , Abdelrahim R , Altihilli K . Pyridoxine responsive seizures: beyond aldehyde dehydrogenase 7A1. J Neurosci Rural Pract. 2019;10:613‐616. 10.1055/s-0039-1697775. 31831980 PMC6906095

[14] Pitt JJ , Eggington M , Kahler SG . Comprehensive screening of urine samples for inborn errors of metabolism by electrospray tandem mass spectrometry. Clin Chem. 2002;48:1970‐1980. 12406983

[15] Sadedin SP , Dashnow H , James PA , et al. Cpipe: a shared variant detection pipeline designed for diagnostic settings. Genome Med. 2015;7:68. 10.1186/s13073-015-0191-x. 26217397 PMC4515933

[16] Tremino L , Forcada‐Nadal A , Rubio V . Insight into vitamin B6‐dependent epilepsy due to PLPBP (previously PROSC) missense mutations. Hum Mutat. 2018;39:1002‐1013. 10.1002/humu.23540. 29689137

[17] Lee S , Chen DY , Zaki MS , et al. Bi‐allelic loss of human APC2, encoding adenomatous polyposis coli protein 2, leads to lissencephaly, subcortical heterotopia, and global developmental delay. Am J Hum Genet. 2019;105:844‐853. 10.1016/j.ajhg.2019.08.013. 31585108 PMC6817548

[18] Mills PB , Camuzeaux SS , Footitt EJ , et al. Epilepsy due to PNPO mutations: genotype, environment and treatment affect presentation and outcome. Brain. 2014;137:1350‐1360. 10.1093/brain/awu051. 24645144 PMC3999720

[19] Mills PB , Footitt EJ , Mills KA , et al. Genotypic and phenotypic spectrum of pyridoxine‐dependent epilepsy (ALDH7A1 deficiency). Brain. 2010;133:2148‐2159. 10.1093/brain/awq143. 20554659 PMC2892945

[20] Alghamdi M , Bashiri FA , Abdelhakim M , et al. Phenotypic and molecular spectrum of pyridoxamine‐5′‐phosphate oxidase deficiency: a scoping review of 87 cases of pyridoxamine‐5′‐phosphate oxidase deficiency. Clin Genet. 2020;1–12. 10.1111/cge.13843. PMC7820968 32888189

[21] Mills PB , Struys E , Jakobs C , et al. Mutations in antiquitin in individuals with pyridoxine‐dependent seizures. Nat Med. 2006;12:307‐309. 10.1038/nm1366. 16491085

[22] Mathis D , Abela L , Albersen M , et al. The value of plasma vitamin B6 profiles in early onset epileptic encephalopathies. J Inherit Metab Dis. 2016;39:733‐741. 10.1007/s10545-016-9955-8. 27342130

[23] Wilson MP , Footitt EJ , Papandreou A , et al. An LC‐MS/MS‐based method for the quantification of pyridox(am)ine 5′‐phosphate oxidase activity in dried blood spots from patients with epilepsy. Anal Chem. 2017;89:8892‐8900. 10.1021/acs.analchem.7b01358. 28782931 PMC5588098

[24] Footitt EJ , Heales SJ , Mills PB , Allen GF , Oppenheim M , Clayton PT . Pyridoxal 5′‐phosphate in cerebrospinal fluid; factors affecting concentration. J Inherit Metab Dis. 2011;34:529‐538. 10.1007/s10545-011-9279-7. 21305354

