# Supplementary material for: Early‐onset vitamin B6‐dependent epilepsy due to pathogenic PLPBP variants in a premature infant: A case report and review of the literature
Source: JIMD Rep. 2020 Nov 15;58(1):3–11. doi: 10.1002/jmd2.12183 (PMC7932866; doi:10.1002/jmd2.12183)
Supplement: Supplementary file 1 — Table S1. Clinical features of patients with early‐onset vitamin B6‐dependent epilepsy caused by variants in PLPBP Table S2. Metabolic profiles of patients with PLPBP variants described to date [file JMD2-58-3-s001.docx]

**Supplementary Table 1. Clinical features of patients with early-onset vitamin B_6_-dependent epilepsy caused by variants in *PLPBP***

| Reference | This study | Darin^4^ | Plecko^5^ | Shiraku^9^ | Johnstone^8^ | Jensen^11^ | Jiao^12^ | Kernohan^10^ | Koul^13^ | Total |
| --- | --- | --- | --- | --- | --- | --- | --- | --- | --- | --- |
| Gender | F | M 4; F 3 | M2; F2 | M4 | M6; F6 | M2 | F2 | NR 1 | NR 12 | M18;F14 (NR13) |
| Current age | 3 m corr. | 4.5 m (D)- 16 y | 26 m – 30 y | 3 y – 8 y | 8 wk (D) – 14 y | 7 wk (D)– 23 m | 17 m – 4y9 m | 58d (D) | NR 12 | 3 m – 30 y  Deaths X5 7 wk - 4.5 m |
| Ethnicity | Australian | Syrian 3  Indian 2  Italian 1  German 1 | Swiss-Italian  German  Arabic  Italian | Japanese 2  Malaysian 2 | Arab 5, Dutch 1, African/Creole 1, African/American 1, Hispanic1, Kurdish 2, Cree First Nation 1 | Turkish  Indian | Chinese 2 | NR 1 | NR 12 | Australian 1, Syrian 3, Arabic 6, Kurdish 2, Turkish 1, Malayisna 2, Japanese 2, Chinese 2, Indian 3, German 2, Italian 2, Swiss-It 1, Dutch 1, Afr-Am 1, Afr-Cr 1, Hisp 1; CFN 1; NR 13 |
| Consanguinity | No | Yes, 5 from 3 families  No 2 | Yes,2 from 2 families  No 2 | Yes, 1 from 1 family  No 3 | Yes, 10 from 9 families  No 1 | Yes, 2 from 2 families | No 2 | NR 1 | NR 12 | Yes, 20  from 17 families  No 12 NR 13 |
| Pregnancy/  delivery complications | Yes | Yes 3  No 4 | No 4 | Yes 1  No 3 | Yes 6  No 6 | No 2 | No 2 | NR 1 | NR 12 | Yes 11/32  No 21/32; NR 13 |
| Foetal distress | Yes | Yes 4  No 3 | NR 4 | No 4 | Yes 3  NR 9 | NR2 | No 2 | NR1 | Yes 2  NR 10 | Yes 10/19; No 9/19  NR 26 |
| Gestational age in weeks | 34+4 (LP) | P 1  LP 3  T 3 | T 4 | P 1  T 3 | LP 1  T 11 | LP 1  T 1 | NR 2 | NR1 | NR 12 | P 2  LP 6  T 22 NR 15 |
| Birth HC centile | 10-25% | <10% 4  25-50% 2  50-90% 1 | 25-50 % 1  50-90%  NR 1 | 10-25% 1  25-50% 3 | < 10% 3  10-25% 3  25-50% 2  50-90% 2  NR 2 | < 10% 2 | NR 2 | NR 1 | NR 12 | < 10% 9/27  10-25% 5/27  25-50% 8/27  50-90% 5/27  NR 18 |
| Acquired microcephaly | Yes | Yes 6, No 1 | No 4 | Yes 2, No 2 | NR 12 | NR 2 | NR2 | NR 1 | NR 12 | Yes 9/16; No 7/16  NR 29 |
| Seizure onset | <24h | <24 h 6  2-7 d  1-4 wk  >1 m 1 | <24 h 0  2-7 d 3  1-4 wk 1  >1m | <24 h 1  2-7 d  1-4 wk 1  >1m 2 | <24 h 5  2-7 d 6  1-4 wk  >1m 1 | <24 h 2  2-7 d  1-4 wk  >1m | <24 h 1  2-7 d  1-4 wk 1  >1m | NR 1 | <24h 3  2-7d  1-4 wk 1  NR 8 | < 24 h 19/36  2-7 d 9/36  1-4 wk 4/36  > 1 m 4/36  NR 9 |
| Seizure type | GTC  Clo  SIA | Ton 4  Clo 1  GTC 5  Myo 2  SIA 3 | Ton 1  Clo 1  Myo 1  SIA 2 | Ton 2  Clo 2  GTC 4  Myo 2  SIA 1 | Ton 5  Clo 2  GTC 3  Myo 4  Spasms 4 | Ton 1  Clo 1  GTC  Myo 1  SIA 2 | Clo 1  GTC 1  Myo 1  SIA  Spasms 1 | NR 1 | GTC 4  Myo 1  NR 8 | Tonic 13/36  Clonic 9/36  GTC 18/36  Myo 12/36  SIA 9/36  Spasms 5/36 NR 9 |
| EEG findings | BS | BS 5  RBA 3 NR3  FD 1; NR 5  MFS 1 NR5 | BS 1  RBA 1  FD 1  MFS 1 | BS 1  RBA 1  FD 1  MFS 2 | BS 6  RBA  FD 1  MFS 2 | BS  RBA  FD 1  MFS 1 | BS  RBA  FD  MFS 1 | NR 1 | BS 2  Normal 2  NR 8 | BS 16/36  RBA 5/36  FD 5/36  MFS 8/36  NR 9 |
| Response to AEDs before vitamin B_6_ | PB, MDZ, LEV (refractory) | Refractory 1  Minimal 1  Partial 2  NA3 | Refractory 3 control 1 | Refractory 1  Partial 3 | Refractory 3  Partial 7  NA 2 | Refractory 2 | Refractory1  Partial 1 | NR 1 | Refractory 12 | Refractory 25/44  Partial 13/44  Good 1/44  NA/NR 6 |
| Initial B_6_ treatment (response/age) | PN  (4d/sz free) | PN 7  (NR/sz free) | PN 4 (sz free)  <1wk 1  1-4 wk 1  > 1m 2 | PN 3; PLP 1  <1wk 0  1-4 wk 1  > 1m 2  (Sz free 1, good 2, Nil 1) | PN 8 (sz free 6, good 1, partial 1)  PLP 1 (good)  PN+PLP 1 (sz free); NA 2 died never treated | PN 1(sz free)  NA 1(Died – never treated) | PN (NR 2) | NA 1 (D – never treated) | PN 12 | PN 38/41; PLP 2/41  PN+PLP 1/41  Died before Rx 4  Sz free 31/39;good ctrl 4/39; partial 1/39; Nil effect 3/39;  NA 4 NR 2 |
| B_6_ vitamer switch (type/response) | PLP (10d)/  sz free | PLP/  sz free 4 | No | No | PLP/  (no improvement 1,  sz free 1)  PN/  (no improvement 1) | PLP 1/ (good)  NA 1 | No 2 | NA 1 | NR 12 | PLP 8   - Sz free 6 - Good ctrl 1 - No improvement 1   PN 1   - No improvement 1 |
| B_6_ withdrawal (sz relapse/ poor response) | No | Yes/ (Yes) 2 | Yes/(Yes) 2  No 2 | Yes/(Yes) 1  No 3 | Yes (PN3, PLP1)  /(Yes) 4  NA 2 | No 1  NA 1 | Yes/(Yes) 2 | NA 1 | Yes/  Yes 12 | Yes/(Yes) 23/41    NA 4 |
| Current treatment (dose) | PLP  (30 mg/kg/d) | PN 2 (30 mg/kg/d)  PLP 4 (30-45 mg/kg/d)    NA 1 (D) | PN 4 (150-450 mg/d) | PLP 1 (200mg/d)  PN 2 (100 mg/d);    NR 1 | PN (5-40 mg/kg/d) 9; PLP (60 mg/kg/d) 1;    NA 2 (D) | PLP 1  30 mg/kg/d    NA 1 (D) | PN 2  (1.6-12.8 mg/kg/d) | NA 1 (D) | PN 10    NR 2 | PN 26/37 (5.7-30-60 mg/kg/d)  PLP 8/37 (2-45 mg/kg/d)  NA 5 (D); NR 3 |
| Current additional AEDs (dose) | LEV  (20 mg/kg/d) | Yes 5  No 1;  NA 1 | VAL (15 mg/kg/d) 1;  No 3 | Yes 3  No1 | Yes 5;  No 5;  NA 2 | LAM (10 mg/kg/d) 1;  NA 1 (D) | No 2 | NA 1 | NR 12 | Yes 16/28;  No 12/28  NA 5 (D); NR 12 |
| Epilepsy course | Sz free | Breakthrough febrile sz 5,  NR 1  NA 1 (D) | Breakthrough febrile sz 1, sporadic afebrile sz 2, photosensitive sz 1 | Sz free 2  Breakthrough febrile sz 1, sporadic afebrile sz 1 | Sz free 3, Breakthrough febrile sz 7,  NA 2 (D) | Breakthrough febrile sz 1  NA 1 (D) | Sz free1  Breakthrough febrile sz 1 | NA 1 (D) | Sz free 10  NR 2 | Sz free 17/37  Breakthrough febrile sz 16/37  Sporadic afebrile sz 3/37  Photosensitive sz 1/37  NA 5 (D); NR 3 |
| Delayed developmental milestones | No | Yes 5  No 1,  NA 1 (D) | Yes 1, Slightly 1, No 2 | Yes 4 (motor & speech) | Yes 5 (motor & speech), No 5,  NA 2 (D) | Yes 1  NA 1 (D) | NR 2 | NA 1 | Yes 2  No 10 | Yes/Slight 19/38  No 19/38  NA 5 (D), NR 2 |
| Intellectual disability | NA | Yes 6  NA 1 | Yes 1  No 2  NR1 | Yes 4 (mild to profound) | poor 1, average 1, excellent 1  NR 6, NA 3 | NA 2 | NR 2 | NA 1 | Yes 1  No3  NR 8 | Yes 13/20  NA 8; NR17 |
| Brain MRI | WM changes  Simplified sulcation  Sub-ependymal cysts  Haemorrhage | Simplified sulcation 4  Periventricular cysts 4  NR 3 | Normal 4 | WM changes 3  Broad gyri/shallow sulci 3  Normal 1 | Normal 4  WM changes 6  Paraventricular cysts 2  Broad gyri 1  PLIC not myelinated 3    NR 1 | Broad gyri/shallow sulci 1  Delayed PLIC myelination 1      NA 1 | Normal 1  NR 1 | NR 1 | WM 1  Normal 2  NR 9 | WM changes 11/29  Simplified Sulcation 10/29  Cysts 7/29  Poor PLIC myelination 3/29  Normal 12/29  NR/NA: 16 |
| Abbreviations: AEDs, antiepileptic drugs; BS, burst suppression; Clo, clonic; D, died; d, days; FD, focal discharges; GTC, generalized tonic-clonic; h, hours; LAM, lamotrigine; LEV, levetiracetam; LP, late-preterm (34^+0^ - 36^+6^ weeks); m, months; MDZ, midazolam; MFS, multifocal spikes; Myo, myoclonic; NA; not applicable; NR, not reported; P, pre-term (<34 weeks); PB, phenobarbitone; PLIC, posterior limb or internal capsule; PLP, pyridoxal-5’-phosphate; PN, pyridoxine; RBA, reduced background activity; SIA, seizures with impaired awareness (lip-smacking, eye deviation); sz, seizure; T, Term (37+0-40 weeks); Ton, tonic; VAL, valproate; wk, weeks; y, years | | | | | | | | | | |

**Supplementary Table 2. Metabolic profiles of patients with *PLPBP* variants described to date**

| Reference | Urine | Plasma | CSF |
| --- | --- | --- | --- |
| **This study**^ƛ^  (*n*=1) | **High Vanil lactic**  **High Pyridoxic acid ^*^**  **Low Guanidino acetic**  **Low Creatine**  Normal pipecolic acid  Normal P6C | **Acidosis**  **High Lactate**  **Anaemia at birth**  Amino acids:   - **High Gly** - **High Ala**   Vitamer profiles: NM | **High lactate**  Amino Acids: NM (protein contamination)  Neurotransmitters:   - **Low HVA** - **Low 5-HIAA** - Normal HVA:5-HIAA ratio - 3-oromethyldopa NM - L-dopa NM - 5-hydroxytryptophan NM   Homocarnosine: NM  Vitamer profiles: NM |
| **Darin^4^**  (*n*=7) | **High Vanil lactic** in 3/4  NR 3 | **Acidosis** in 5/7  **High lactate** in 4/7  **Anaemia at birth** in 2/7  Amino acids in 2/2   - **High Gly** - **High Ala**   Vitamer profiles (on treatment)::   - **High PLP** in 3/3 | Lactate: NR  Amino Acids   - **High Gly** in 2/2 - **High Ala** in 1/1   Neurotransmitters:   - **Low HVA** in 1/4 - Normal 5-HIAA in 4/4 - **High 3-oromethyldopa** in 2/3 - **High L-dopa** in 1/2 - **High 5-hydroxytryptophan** in 2/2   Homocarnosine: **undetectable** in 1/1  Vitamer profiles:   - **Low PLP** in 2/3 (pre-treatment) - **Low PL** in 1/1 (pre-treatment) |
| **Plecko^5^**  (*n*=4) | Normal in 1  NR 3 | Acidosis: NR  Lactate: NR  Anaemia at birth in 0/2  Amino acids in 1/2   - **High Gly** - **High Ala**   Vitamer profiles (on treatment):   - **High PLP** in 4/4 - **High PL** in 4/4 - **High PA** in 3/4 | Lactate: NR  Amino Acids in 1/1   - **High Gly** - **High Ala**   Neurotransmitters in 1/1   - Normal HVA (on treatment) - Normal 5-HIAA (on treatment) - Normal 3-oromethyldopa - Normal L-dopa - Normal 5-hydroxytryptophan   Homocarnosine: NR  Vitamer profiles: NR |
| **Shiraku^9^**  (*n*=4) | Normal in 1  NR 3 | **Acidosis** in 1/4  **High lactate** in 1/4  Anaemia at birth: NR  Amino acids   - **High Gly** in 1/3 - **High Thr** in 1/3   Vitamer profiles: NR | Lactate: NR  Amino Acids in 1/1   - **High Gly** - **High Thr** - Normal Gly ratio   Neurotransmitters: NR  Homocarnosine: NR  Vitamer profiles: NR |
| **Johnstone^8^**  (*n*=12) | **High Vanil lactic** in 1/7  **High Vanil pyruvic** in 1/7  **High N-acetylvanilalanine** in 1/7  Minor elevations in urine lactic, pyruvic and 2-OH butyric in 1/7  Minor elevations in malic, 2-ketoglutaric and N-acetylaspartic in 1/7  Mildly elevated AASA in 1/7  Normal in 5/7  NR 5 | **Acidosis** in 2/12  **High lactate** in 6/12  Anaemia at birth: NR  Amino acids   - **High Gly** in 3/12 | **High Lactate** in 1/5  Amino Acids:   - **High Gly** in 2/5 - **High Trp** in 1/5   Neurotransmitters:   - **High 3-methoxytyrosine** in 1/4 - Normal in 3/4   Homocarnosine: NR  Vitamer profiles: NR |
| **Kernohan^10^**  (*n*=1) | NR 1 | NR 1 | NR 1 |
| **Jensen^11^**  (*n*=2) | **Lactic acid** in 1/2  Nomal in 1/2 | **Acidosis** in 2/2  **High lactate** in 2/2  Anaemia at birth in 0/2  Amino acids   - **High Gly** in 2/2 | Lactate: NR  Amino Acids:   - **High Gly** in 1/2 - **High Tyr** in 1/2 - Normal Trp in 1   Neurotransmitters: NR  Homocarnosine:   - normal in 1   Vitamer profiles: NR |
| **Jiao^12^**  (*n*=2) | NR 2 | Acidosis: NR  **High lactate** in 2/2  Anaemia at birth: NR  Amino acids: NR | NR 2 |
| **Koul^13^**  (*n*=12) | Normal pipecolic acid in 4  Normal AASA in 4  NR 8 | NR 12 | NR 12 |
| ^†^Data compiled from cases described in Darin *et al.*^4^, Plecko *et al.*^5^, Johnstone *et al.*^8^, Shiraku *et al.*^9^, Jensen *et al.*^11^, Koul *et al.*^13^ and current study. Unless otherwise specified, results relate to specimens collected before commencement of vitamin B_6_ treatment.  ^ƛ^The rest of this patient’s metabolic investigations were normal including acylcarnitine profile, biotinidase assay, very long chain fatty acids, phytanic acid, plasmalogens, transferrin isoforms and CSF pterins.  ^*^Urine specimen in our patient was collected 24 hours after commencement of pyridoxine treatment.  Abbreviations: CSF, cerebrospinal fluid; P6C, Δ1-piperideine-6-caboxylate; Ala, alanine; Gly, glycine; Thr, threonine; Trp, tryptophan; Tyr, tyrosine; PL, pyridoxal; PLP, pyridoxal-5'-phosphate; PA, pyridoxic acid; NM, not measured; NR, not reported | | | |
